# Noise-Robust Image Reconstruction Based on Minimizing Extended Class of Power-Divergence Measures

**DOI:** 10.3390/e23081005

**Published:** 2021-07-31

**Authors:** Ryosuke Kasai, Yusaku Yamaguchi, Takeshi Kojima, Omar M. Abou Al-Ola, Tetsuya Yoshinaga

**Affiliations:** 1 Graduate School of Health Sciences, Tokushima University, 3-18-15 Kuramoto, Tokushima 770-8509, Japan; kasai-r@tokushima-u.ac.jp; 2 Shikoku Medical Center for Children and Adults, National Hospital Organization, 2-1-1 Senyu, Zentsuji 765-8507, Japan; yamaguchi.yusaku.sf@mail.hosp.go.jp; 3 Institute of Biomedical Sciences, Tokushima University, 3-18-15 Kuramoto, Tokushima 770-8509, Japan; kojima@medsci.tokushima-u.ac.jp; 4 Faculty of Science, Tanta University, El-Giesh Street, Tanta 31527, Egypt; omar26_7@yahoo.com

**Keywords:** power-divergence measure, computed tomography, iterative reconstruction, maximum-likelihood expectation-maximization method, continuous-time image reconstruction

## Abstract

The problem of tomographic image reconstruction can be reduced to an optimization problem of finding unknown pixel values subject to minimizing the difference between the measured and forward projections. Iterative image reconstruction algorithms provide significant improvements over transform methods in computed tomography. In this paper, we present an extended class of power-divergence measures (PDMs), which includes a large set of distance and relative entropy measures, and propose an iterative reconstruction algorithm based on the extended PDM (EPDM) as an objective function for the optimization strategy. For this purpose, we introduce a system of nonlinear differential equations whose Lyapunov function is equivalent to the EPDM. Then, we derive an iterative formula by multiplicative discretization of the continuous-time system. Since the parameterized EPDM family includes the Kullback–Leibler divergence, the resulting iterative algorithm is a natural extension of the maximum-likelihood expectation-maximization (MLEM) method. We conducted image reconstruction experiments using noisy projection data and found that the proposed algorithm outperformed MLEM and could reconstruct high-quality images that were robust to measured noise by properly selecting parameters.

## 1. Introduction

Image reconstruction in computed tomography (CT) is the process of estimating unknown density images from measured projections. When the system of a tomographic inverse problem is ill-posed, iterative reconstruction algorithms [1,2] based on the optimization strategy provide significant improvements over transform methods, including the filtered back-projection [3,4] (FBP) procedure. In recent years, iterative reconstruction has received much attention because of its ability to reduce radiation doses [5,6,7,8,9] in X-ray CT. Iterative algorithms implemented in, e.g., the algebraic reconstruction technique [1], maximum-likelihood expectation-maximization [10] (MLEM) method, and multiplicative algebraic reconstruction technique, have been used for reconstructing CT images. The MLEM algorithm, which is the most popular method used in emission CT and is derived for the maximum likelihood estimation of a Poisson distribution, reconstructs high-quality images even for noisy projection data, but it is slow to converge [11,12,13,14] under iteration. The ordered-subsets EM algorithm [11], in which the EM iteration is performed in each subset by dividing the projection into subsets or blocks, is known to be useful for accelerating MLEM [13,15,16]. However, divergence or oscillation of solutions may occur in the iterative process when the subset balance is not satisfied. Because of the high quality of image reconstruction afforded by the MLEM algorithm, improved MLEM methods have been presented for accelerating convergence. Some schemes accelerate the convergence rate by increasing a relaxation parameter or the step-size in iterative operations [14,17,18] or by introducing a parameter with a power exponent related to the projection for controlling the noise model [19,20]. However, no theory has explained the divergence and oscillation phenomena affecting solutions when the step-size parameter is large.

The convergence of iterative solutions and the quality of images are governed by the underlying objective function that has to be minimized. Hence, the base objective function is one of the most important decisions when designing an iterative algorithm. In this paper, we present an extended class of power-divergence measures [21,22,23,24] (PDMs) and derive a novel iterative algorithm based on the minimization of the extended PDM (EPDM) as an objective function for the optimization strategy. Let us define the parameterized function $\Phi_{\gamma ,\alpha }\left(p,q\right)$ of vectors *p* and *q* with nonnegative elements $p_{i}$ and $q_{i}$, respectively, as

(1) $$\Phi_{\gamma ,\alpha }\left(p,q\right):=\sum_{i}\int_{p_{i}}^{q_{i}}\frac{s^{\gamma }-p_{i}^{\gamma }}{s^{\gamma \alpha }}ds$$

where γ and α indicate positive and nonnegative parameters, respectively. The extension is performed by incorporating the parameter α in the conventional class of PDMs, which includes a large set of distance and relative entropy measures. By fixing the parameter α=1, $\Phi_{\gamma ,1}$ gives the family of PDMs with a single parameter γ. Therefore, the measure coincides with the Kullback–Leibler (KL), or relative entropy, divergence [25] if γ=1, Neyman’s $\chi^{2}$ distance if γ=2, and the generalized Hellinger distance otherwise. Moreover, it corresponds to the squared $L^{2}$ norm when γ=1 and α=0 and the reverse KL-divergence when γ=1 and α=2. Thus, the parameters γ and α provide a smooth connection among the forward and reverse KL-divergences, the Hellinger distance, the $\chi^{2}$ distance, and the $L^{2}$ distance and can control the trade-off between robustness and asymptotic efficiency of the estimators, in a similar way as in other families of distance measures [26,27,28,29].

By exploiting the vectors *p* and *q* in Equation (1) as the measured and forward projections, respectively, for the tomographic inverse problem, it is expected that we can create a high-performance iterative reconstruction algorithm thanks to the high degree of freedom. For constructing this novel iterative algorithm, we introduce a nonlinear differential equation whose numerical discretization is equivalent to the iterative system. The purpose of applying a dynamical method [30,31,32,33,34,35] to tomographic inverse problems [36,37,38,39] is as follows: it enables us to prove the stability of the equilibrium corresponding to the desired solution of the system of differential equations by using the Lyapunov stability theorem [40] if a proper Lyapunov function can be found; then, since the step-size used to discretize the set of differential equations corresponds to the relaxation or scaling parameter of the system of difference equations, a family of iterative algorithms incorporating a parameter for acceleration is naturally derived. Moreover, it provides a methodology for systematically designing a new iterative reconstruction algorithm based on optimization of an objective function depending on the features of the image to be reconstructed.

Since the EPDM family includes the KL-divergence, the resulting iterative algorithm with power exponents corresponding to the parameters γ and α is a natural extension of the MLEM method with (γ,α)=(1,1). The convergence of solutions to the continuous analog of the proposed iterative algorithm is theoretically shown using the EPDM as a Lyapunov function when the tomographic inverse problem is consistent.

We conducted image reconstruction experiments using numerical and physical phantoms with noisy projections and found that the proposed algorithm outperformed the conventional MLEM method with respect to reconstructing high-quality images that are robust to measured noise when selecting a set of proper parameter values.

## 2. Definitions and Notations

Image reconstruction is a problem of obtaining unknown pixel values $x\in R_{+}^{J}$ satisfying

(2) y=Ax+δ

where $y\in R_{+}^{I}$, $A\in R_{+}^{I\times J}$, and $\delta \in R^{I}$ denote the measured projection, projection operator, and noise, respectively, with $R_{+}$ representing the set of nonnegative real numbers. When the system in Equation (2) without noise, i.e., δ=0, has a solution $e\in R_{+}^{J}$, it is consistent; otherwise, it is inconsistent. The tomographic inverse problem can be reduced to one of finding *x*, which can be accomplished using an optimization approach such as an iterative method or a continuous-time system by minimizing an objective function.

Here, we introduce the notation that will be used below. The superscript ⊤ stands for the transpose of a matrix or vector, $\theta_{k}$ indicates the *k*th element of the vector θ, $\Theta_{i}$ and $\Theta_{ij}$ indicate the *i*th row vector and the element in the *i*th row and *j*th column of the matrix Θ, respectively, Log(θ) and Exp(θ) are, respectively, the vector-valued functions $\mathrm{Log}\left(\theta \right):={\left(\log\left(\theta_{1}\right),\log\left(\theta_{2}\right),\ldots ,\log\left(\theta_{i}\right)\right)}^{⊤}$ and $\mathrm{Exp}\left(\theta \right):={\left(\exp\left(\theta_{1}\right),\exp\left(\theta_{2}\right),\ldots ,\exp\left(\theta_{i}\right)\right)}^{⊤}$ of each element in vector $\theta ={\left(\theta_{1},\theta_{2},\ldots ,\theta_{i}\right)}^{⊤}$, and diag(θ) indicates the diagonal matrix in which the diagonal entries are the elements of the vector θ.

## 3. Proposed System

### 3.1. Definition

The proposed methods for obtaining a solution to the tomographic inverse problem can be described as an iterative algorithm and dynamical system.

We present an iterative reconstruction method with a relaxation or scaling parameter h>0:

(3) $$z_{j}\left(n+1\right)=z_{j}\left(n\right){\left(f_{j}\left(z\left(n\right)\right)\right)}^{h}$$

with

(4) $$f_{j}\left(w\right):=\frac{\sum_{i=1}^{I}A_{ij}{\left(\frac{y_{i}}{{\left(A_{i}w\right)}^{\alpha }}\right)}^{\gamma }}{\sum_{i=1}^{I}A_{ij}{\left(\frac{A_{i}w}{{\left(A_{i}w\right)}^{\alpha }}\right)}^{\gamma }}$$

for j=1,2,…,J and n=0,1,2,…,N−1, where γ>0, α≥0, and $z\left(0\right)=z^{0}\in R_{++}^{J}$, with $R_{++}$ representing the set of positive real numbers. The accompanying system derived from a continuous analog based on the dynamical method is described by a dynamical system:

(5) $$\frac{dx_{j}\left(t\right)}{dt}=x_{j}\left(t\right)\log\left(f_{j}\left(x\left(t\right)\right)\right)$$

for j=1,2,…,J at t≥0, where the function $f_{j}$ is in Equation (4) and $x\left(0\right)=z^{0}$. The system in Equation (5) can be equivalently written as

(6) $$\begin{array}{ccc} \frac{dx(t)}{dt} & = & X\left(\mathrm{Log}(A^{⊤}\mathrm{Exp}\left(\gamma \left(\mathrm{Log}\left(y\right)-\alpha \mathrm{Log}\left(Ax\left(t\right)\right)\right)\right)\right)\\ & & \;\left(-\mathrm{Log}(A^{⊤}\mathrm{Exp}\left(\gamma \left(1-\alpha \right)\mathrm{Log}\left(Ax\left(t\right)\right)\right)\right) \end{array}$$

where X:=diag(x). The iterative formula in Equation (3) is obtained by discretizing the differential equation of Equation (5) by using the multiplicative Euler method [41,42] with a step-size of *h*. Note that the iterative formula in Equation (3) with h=1 is equivalent to the algorithm presented by Zeng [19] when γ=1, to the algorithm in Reference [20] when α=1, and to the MLEM algorithm when (γ,α)=(1,1).

We apply the proposed divergence in Equation (1) to the tomographic objective function consisting of measured and forward projections. Namely, we define

(7) $$V\left(x\right):=\Phi_{\gamma ,\alpha }\left(y,Ax\right)=\sum_{i=1}^{I}\int_{y_{i}}^{A_{i}x}\frac{s^{\gamma }-y_{i}^{\gamma }}{s^{\gamma \alpha }}ds$$

which can be written as

$$\begin{array}{ccc} V(x) & = & \sum_{i=1}^{I}\int_{y_{i}}^{A_{i}x}\frac{s^{\gamma }-y_{i}^{\gamma }}{s}ds\\ & = & \frac{1}{\gamma }\sum_{i=1}^{I}y_{i}^{\gamma }\left(\log{\left(\frac{y_{i}}{A_{i}x}\right)}^{\gamma }+{\left(\frac{A_{i}x}{y_{i}}\right)}^{\gamma }-1\right) \end{array}$$

if γα=1;

$$\begin{array}{ccc} V(x) & = & \sum_{i=1}^{I}\int_{y_{i}}^{A_{i}x}\frac{s^{\gamma }-y_{i}^{\gamma }}{s^{1+\gamma }}ds\\ & = & \frac{1}{\gamma }\sum_{i=1}^{I}\log{\left(\frac{A_{i}x}{y_{i}}\right)}^{\gamma }+{\left(\frac{y_{i}}{A_{i}x}\right)}^{\gamma }-1 \end{array}$$

if γα=1+γ; and

$$\begin{array}{ccc} V(x) & = & \sum_{i=1}^{I}\int_{y_{i}}^{A_{i}x}\frac{s^{\gamma }-y_{i}^{\gamma }}{s^{\gamma \alpha }}ds\\ & = & \sum_{i=1}^{I}\frac{1}{1-\gamma \alpha }y_{i}^{1+\gamma (1-\alpha )}\left(1-{\left(\frac{A_{i}x}{y_{i}}\right)}^{1-\gamma \alpha }\right)\\ & & \;+\frac{1}{1+\gamma (1-\alpha )}y_{i}^{1+\gamma (1-\alpha )}\left({\left(\frac{A_{i}x}{y_{i}}\right)}^{1+\gamma (1-\alpha )}-1\right) \end{array}$$

otherwise.

### 3.2. Theoretical Results

This section provides a theoretical result on the dynamical system defined in the preceding section. We show that any solution to the continuous analog converges to the desired solution of the system in Equation (2) with δ=0 when the inverse problem is consistent.

**Theorem** **1.** *Assume there exists $e\in R_{++}^{J}$ satisfying y=Ae. Then, e is an equilibrium observed in the continuous-time system in Equation (6) and is asymptotically stable.*

**Proof**  (Proof). We see that *e* is an equilibrium of the system and the solutions to the system are in $R_{++}^{J}$ because the initial state value at t=0 belongs to $R_{++}^{J}$ and the flow cannot pass through the invariant subspace $x_{j}=0$ for j=1,2,…,J in the state space according to the uniqueness of solutions for the initial value problem. The nonnegative function V(x) of $x_{j}>0$ in Equation (7) is well-defined as a candidate of a Lyapunov function. Then, we have the derivative of *V* along the solutions to Equation (6):

(8) $$\begin{array}{ccc} {\left.\frac{dV}{dt}\left(x\right)\right|}_{\left(6\right)} & = & -\sum_{i=1}^{I}\left({\left(\frac{y_{i}}{{\left(A_{i}x\right)}^{\alpha }}\right)}^{\gamma }-{\left(A_{i}x\right)}^{\gamma (1-\alpha )}\right)A_{i}\frac{dx}{dt}\\ & = & -{\left(\xi -\zeta \right)}^{⊤}X\left(\mathrm{Log}\left(\xi \right)-\mathrm{Log}\left(\zeta \right)\right)\\ & \leq & 0 \end{array}$$

where

$$\begin{array}{ccc} \xi & := & A^{⊤}\mathrm{Exp}\left(\gamma \left(\mathrm{Log}\left(y\right)-\alpha \mathrm{Log}\left(Ax\right)\right)\right)\\ \zeta & := & A^{⊤}\mathrm{Exp}\left(\gamma \left(1-\alpha \right)\mathrm{Log}\left(Ax\right)\right). \end{array}$$

Therefore, *V* is a Lyapunov function and the equilibrium *e* is asymptotically stable.    □

This theorem guarantees that the proposed difference system in Equation (3) as a first-order approximation to the differential equation in Equation (6) has a stable fixed point *e* when the chosen step-size *h* is sufficiently small to ensure numerical stability.

## 4. Experimental Results and Discussion

We will illustrate the effectiveness of the extended MLEM algorithm based on the EPDM family in Equation (3) with the parameter set (γ,α) (in what follows, the iterative algorithm except for MLEM with (γ,α)=(1,1) is referred to as PDEM) by using examples from numerical and physical CT experiments. The proposed systems were executed using a 6-core processor and computing tools provided by MATLAB (MathWorks, Natick, MA, USA).

We set h=1 and a constant initial value $z_{j}^{0}$ for j=1,2,…,J. Note that variation of *h* is out of the scope of this paper, although setting h>1 would accelerate convergence. In addition, in the numerical simulation, the PDEM algorithm in Equation (3) with h=1 as a simple forward Euler discretization qualitatively approximates the solutions to the differential equation in Equation (6), which were calculated by a standard MATLAB ODE solver ode113 implementing a variable step-size variable order method.

### 4.1. Reconstruction Using Numerical Phantom

**Figure 1:**
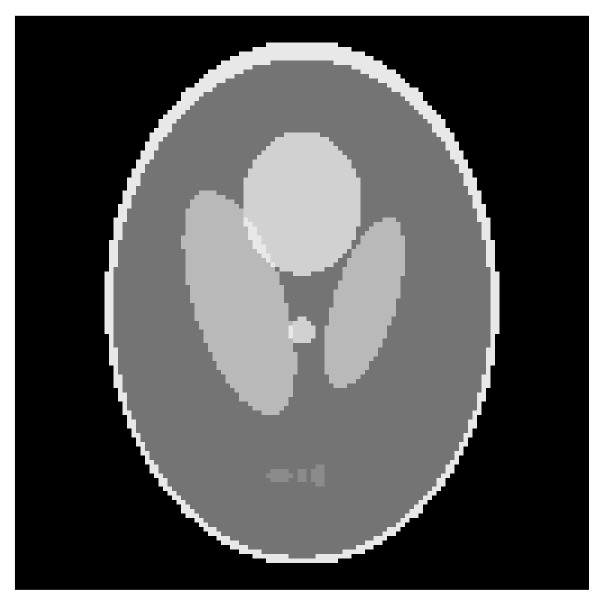
Image of numerical phantom.

We used a numerical phantom image consisting of $e\in {\left[0,1\right]}^{J}$ with 128×128 pixels (J=16,384), as shown in Figure 1. For our experiment, a Shepp–Logan phantom [43], which is a popular test image for developing reconstruction algorithms, was modified by changing the density values for ellipses so that the resulting image had better visual perception with high contrast. The noise-free and noisy projections $y\in R_{+}^{I}$ derived from the phantom image were, respectively, simulated using Equation (2) without and with δ denoting white Gaussian noise such that the signal-to-noise ratio (SNR) was 30 dB and by setting the number of view angles and detector bins to 180 and 184 (I=33,120) with 180-degree sampling.

For directly evaluating the quality of the reconstructed images, we defined functions for comparing the reconstructed image compared against the true image, *e*, as

(9) $$D_{j}\left(z\right):=\left|e_{j}-z_{j}\right|$$

for j=1,2,…,J and

(10) $$E\left(z\right):=||e-{z||}_{2}={\left(\sum_{j=1}^{J}{\left(D_{j}\left(z\right)\right)}^{2}\right)}^{\frac{1}{2}}.$$

**Figure 2:**
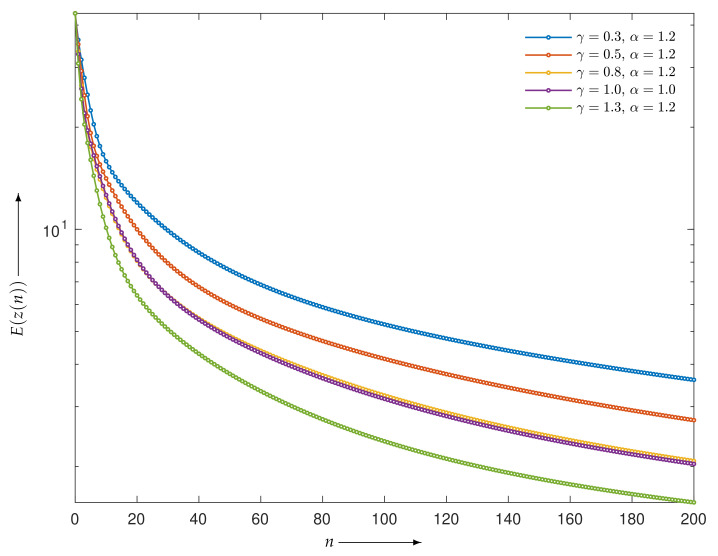
Evaluation functions for MLEM and PDEM algorithms at each iteration in the experiment using numerical phantom with noise-free projection. Note that because the values of PDEM with (γ,α)=(0.8,1.2) and MLEM are very similar, the plotted points for PDEM are almost invisible.

First, we considered the case of a noise-free projection. Figure 2 shows the evaluation functions E(z(n)) of the iterative points z(n) for MLEM and PDEM with the sets of parameters (γ,α) being (0.3,1.2), (0.5,1.2), (0.8,1.2), and (1.3,1.2) for n=0,1,2,…,200. All algorithms monotonically decreased the evaluation function, as supported by the theoretical result that the solutions of the continuous analog converge to the true value. Indeed, another experiment confirmed that the monotonic decrease continued as the number of iterations exceeded 200 iterations. We can see that PDEM with the parameter set (1.3,1.2) reduces the evaluation function much more than MLEM does. To put it another way, the PDEM algorithm takes less computation time than MLEM for obtaining the same evaluation values.

**Figure 3:**
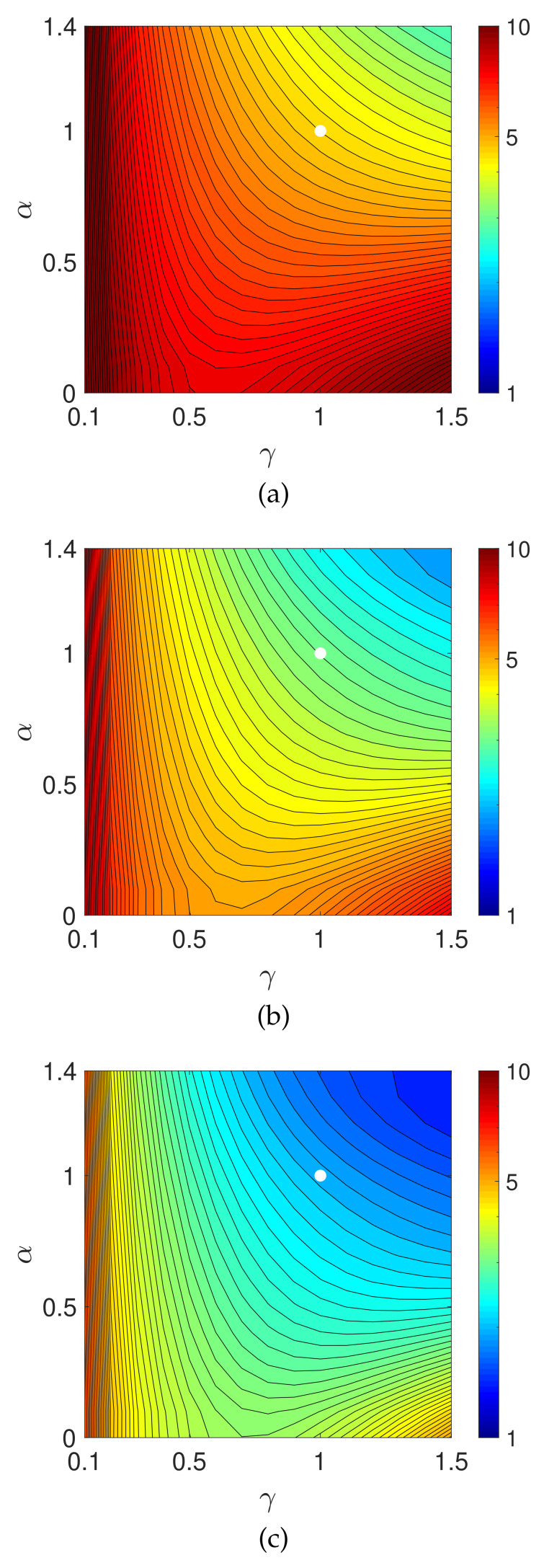
Contour plots of evaluation functions $\log_{10}\left(E\left(z\left(N\right)\right)\right)$ with *N* being (**a**) 50, (**b**) 100, and (**c**) 200 using numerical phantom with noise-free projections. The white dot indicates the position of MLEM.

Figure 3 shows contour plots of the evaluation values on a logarithmic scale, $\log_{10}\left(E\left(z\left(N\right)\right)\right)$ for N=50, 100, and 200, in the parameter plane (γ,α). The parameters γ and α were, respectively, sampled from 0.1 to 1.5 and 0 to 1.4 with a sampling interval of 0.1. We can see that, at least in the range examined, the evaluation function becomes smaller as the values of γ≥1 and α≥1 increase.

**Figure 4:**
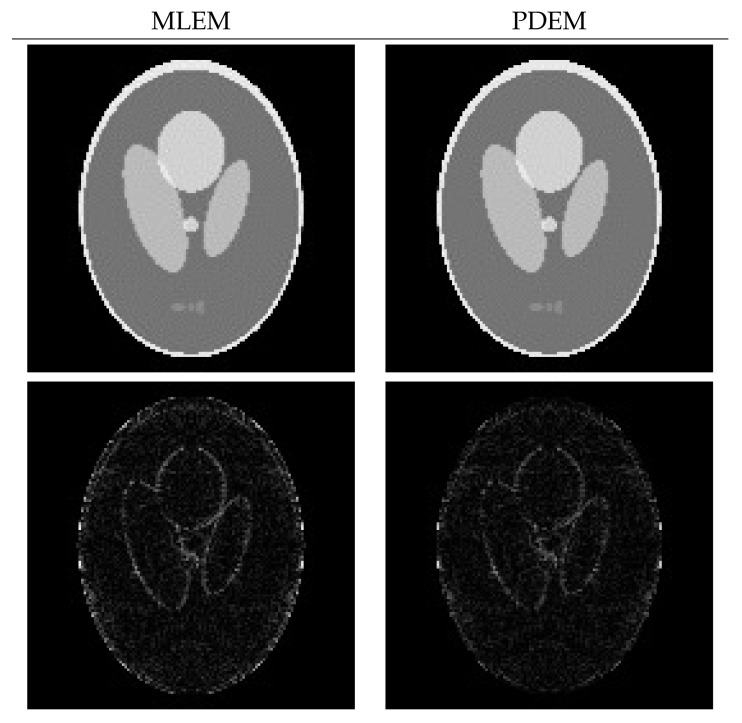
Reconstructed images (**upper panel**) and images of the subtraction (**lower panel**) for MLEM and PDEM with (γ,α)=(1.3,1.2) at 200th iteration using numerical phantom with noise-free projection.

Figure 4 illustrates images reconstructed by MLEM and PDEM with (γ,α)=(1.3,1.2) at the 200th iteration and the corresponding subtraction images $D_{j}\left(z\left(200\right)\right)$ (displayed in the range from 0 to 0.2) at every *j*th pixel, for j=1,2,…,J. By comparing the values of the subtraction between MLEM and PDEM, e.g., the edges of the high-density objects in the image, we can see that PDEM produces high-quality reconstructions, as is quantitatively indicated by its small evaluation value between the reconstructed and phantom images.

**Figure 5:**
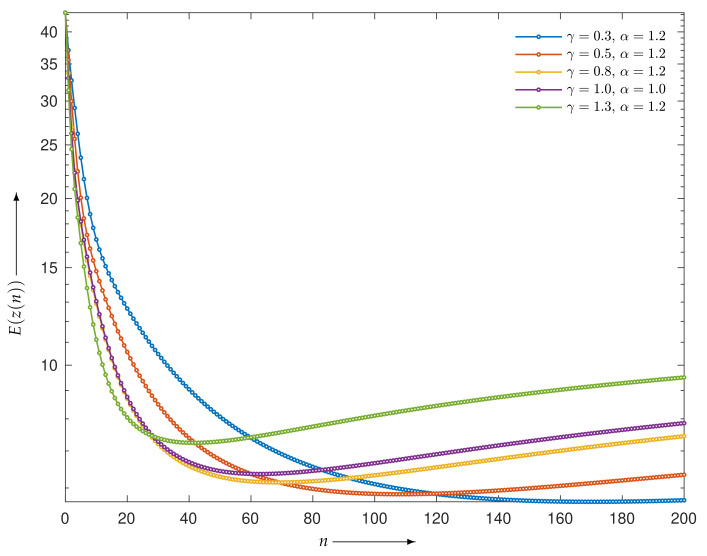
Evaluation functions for MLEM and PDEM algorithms at each iteration in the experiment using numerical phantom with noisy projection.

**Figure 6:**
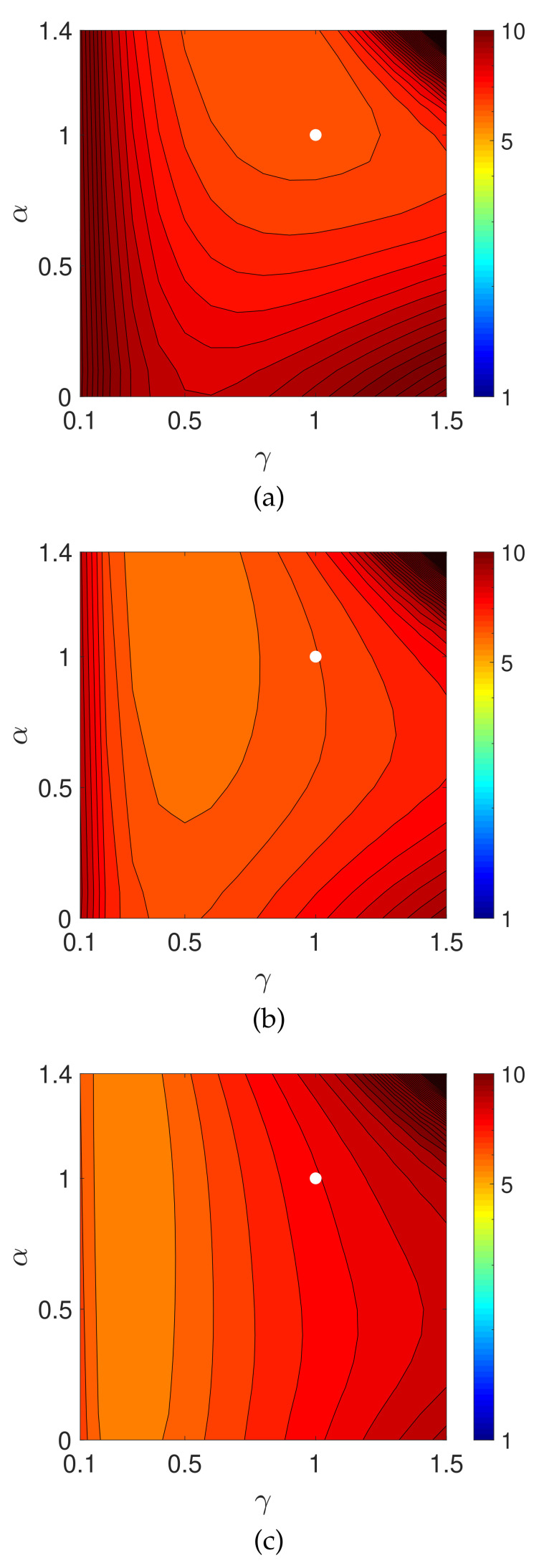
Contour plots of evaluation functions $\log_{10}\left(E\left(z\left(N\right)\right)\right)$ with *N* equal to (**a**) 50, (**b**) 100, and (**c**) 200 in the experiment using numerical phantom with noisy projection. The white dot indicates the position of MLEM.

**Figure 7:**
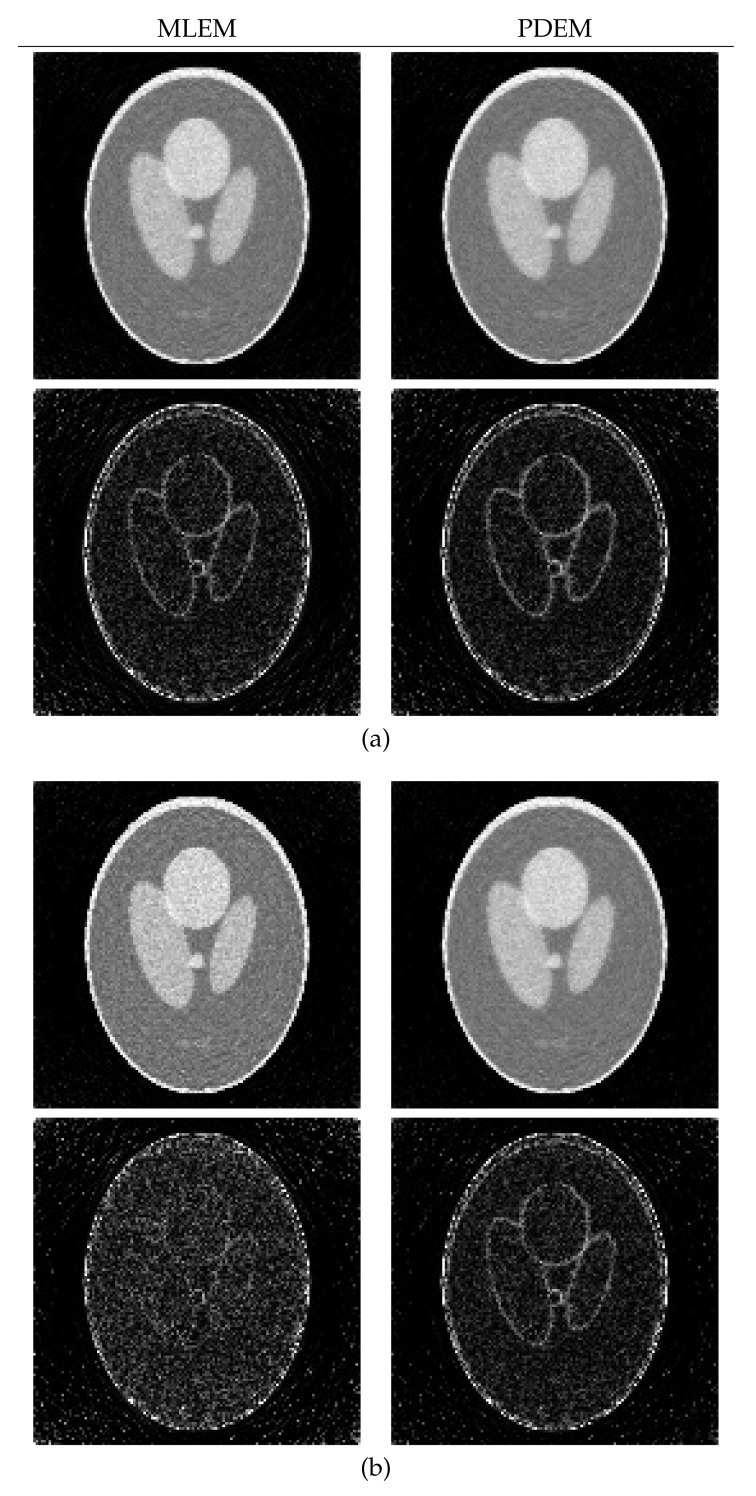

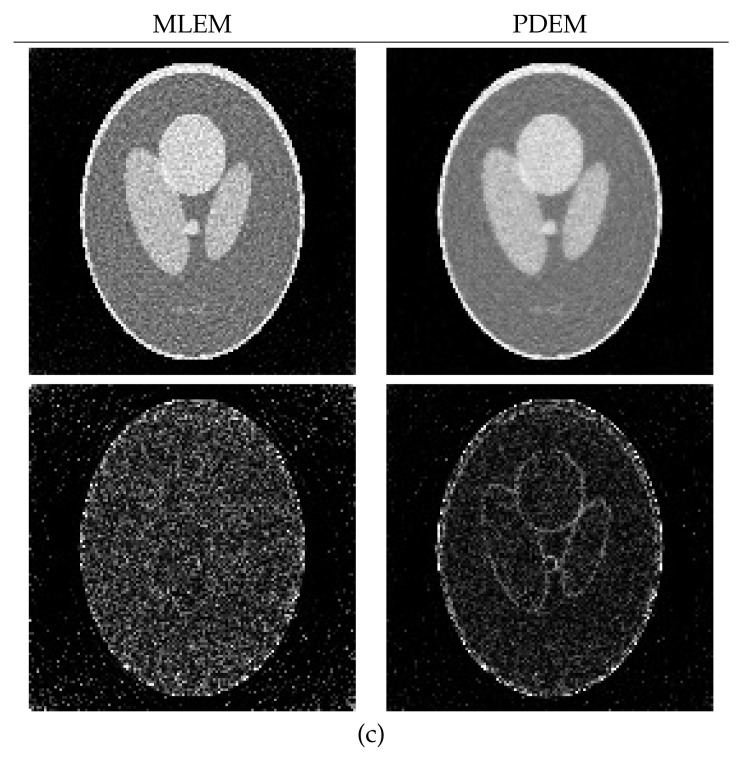
Reconstructed images (**upper panel**) and subtraction images (**lower panel**) for MLEM and PDEM with (γ,α) equal to (**a**) (0.8,1.2) at 50th iteration, (**b**) (0.5,1.2) at 100th iteration, and (**c**) (0.3,1.2) at 200th iteration in the experiment using numerical phantom with noisy projection.

Next, let us consider the effect of the measured noise. Figure 5 is a graph of the evaluation E(z(n)) as a function of iteration number *n* with n=0,1,2,…,200. Given noisy projection data, the algorithm with each parameter set decreases the evaluation function in the early iterations. However, the time course does not show a monotonic decrease in further iterations. Similar characteristics are known to exist and have been considered for the alternative MLEM [19] that is described as Equation (3) with γ=1. We can see that a set of parameters (γ,α) exists at which the PDEM algorithm reduces the evaluation function more than MLEM does for any iteration number. Additionally, the smallest value of the evaluation function among the iteration numbers for a fixed set of the parameters becomes smaller with decreasing γ in the set {0.3,0.5,0.8,1,1.3} considered for this example. The parameter dependence of the evaluation function is clearly visible in contour plots of Figure 6, showing the values of $\log_{10}\left(E\left(z\left(N\right)\right)\right)$ for N=50, 100, and 200 in the parameter plane. When designing a parameterized PDEM algorithm, a relatively large value of α and a small value of γ compared with the reference values of (γ,α)=(1,1) provide the best performance in early and sufficient iterations, respectively. The best choices of (γ,α) depending on the termination iteration number are approximately (0.8,1.2) at the 50th iteration, (0.5,1.2) at the 100th iteration, and (0.3,1.2) at the 200th iteration. The evaluation values under these conditions are indicated in Table 1, showing that PDEM with each parameter set gives a smaller value than MLEM does. The reconstructed images and subtraction images (displayed in the range from 0 to 0.3) are shown in Figure 7. The figure reveals lots of artifacts in the reconstructed image due to the presence of noise in the measured projection. In terms of a quantitative evaluation, the structural similarity index measure [44] (SSIM) between the reconstructed and the true images was calculated and summarized in Table 2. A higher value of SSIM, which is a perception-based quality metric, provides higher image quality. By comparing the images reconstructed by MLEM and PDEM at the 100th and 200th iterations (see Figure 7 and Table 2), we can see that the PDEM with a proper set of parameters is able to reconstruct high-quality images while reducing the effects of noise in the projections, which means that PDEM is more robust to noise than MLEM.

### 4.2. Reconstruction Using Physical Phantom

**Figure 8:**
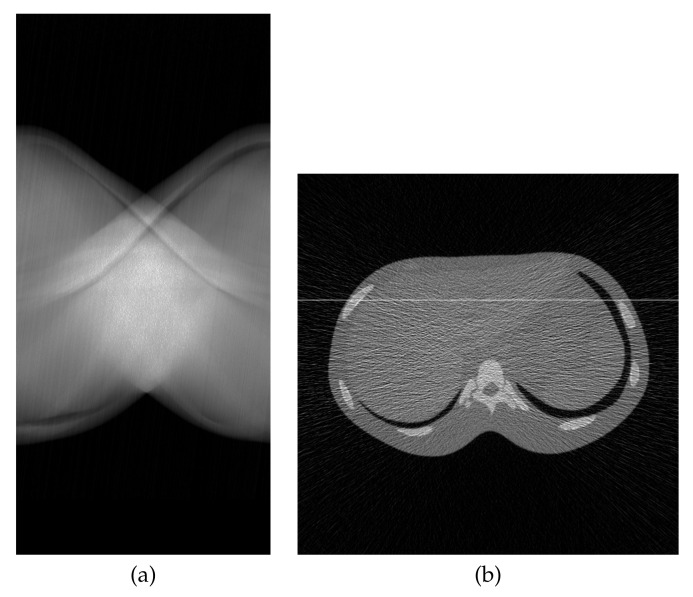
(**a**) Sinogram and (**b**) reconstructed image by FBP in the experiment using physical phantom.

**Figure 9:**
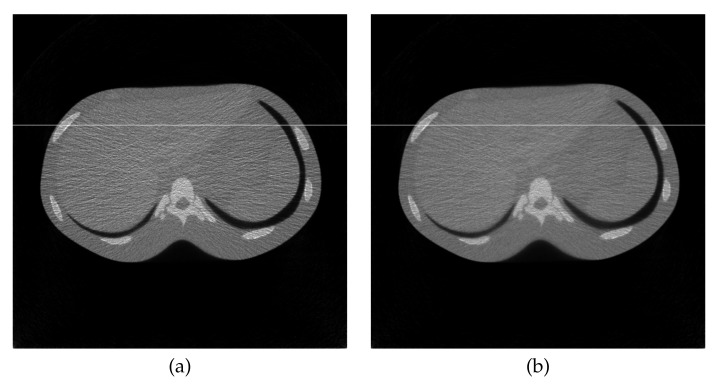
Reconstructed images for (**a**) MLEM and (**b**) PDEM with (γ,α)=(0.5,1.2) at 200th iteration in the experiment using physical phantom.

**Figure 10:**
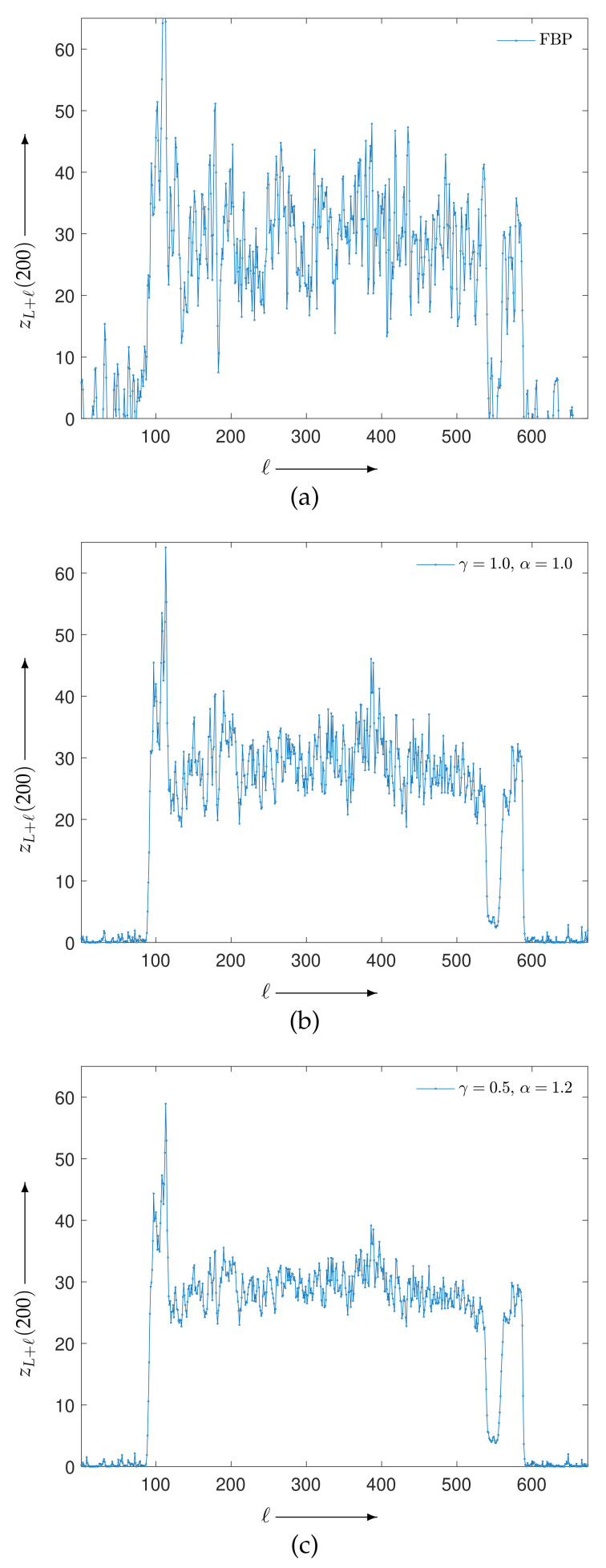
Density profiles for (**a**) FBP, (**b**) MLEM, and (**c**) PDEM of reconstructed images along horizontal line with L=674×224 and ℓ=1,2,…,674.

A physical experiment was carried out to further validate the effectiveness of the proposed method, although the true image is not available for a quantitative evaluation. The projections were physically acquired from an X-ray CT scanner (Canon Medical Systems, Tochigi, Japan) with a body-simulated phantom [45] (Kyoto Kagaku, Kyoto, Japan) using 80 kVp tube voltage, 30 mA tube current, and an exposure time of 0.75 s per rotation. Figure 8 represents the sinogram, a two-dimensional array of data containing the projections $y\in R_{+}^{I}$, with I=430,200 (956 acquisition bins and 450 projection directions in 180 degrees) and a reconstructed image created by FBP using a Shepp–Logan filter with J=454,276 (674×674 pixels). Images reconstructed by MLEM and PDEM with (γ,α)=(0.5,1.2) are shown in Figure 9. The parameter values were referred to as the results of the numerical phantom with noisy projection. Figure 10, which shows the density profiles along horizontal lines (indicated by white) in the reconstructed images of Figure 8b and Figure 9, verifies that the PDEM has a lower density deviation on a flat distribution of the X-ray absorption in the physical phantom than either MLEM or FBP. The parameter values of the power exponents in the PDEM algorithm make it more robust to noise in spite of the higher noise level due to the low-dose X-ray exposure condition. This fact implies that the proposed method contributes to reducing patient doses during X-ray CT examinations in clinical practice by adjusting the parameter values depending on the noise levels of the projection data.

## 5. Conclusions

We presented an extension of the PDM family with two parameters, γ and α, and proposed a novel iterative algorithm based on minimization of the divergence measure as an objective function of the reconstructed images. The theoretical results show the convergence of solutions to the continuous analog of the iterative algorithm owing to the objective function decreasing as the time proceeds. Numerical experiments illustrated that the proposed algorithm, which is considered to be an extended MLEM with two power exponents γ and α, has advantages over MLEM, which is the most popular and suitable iterative method of image reconstruction from noisy measured projections. The algorithm is of practical importance because its image quality is superior to that of MLEM. Our results suggest that a larger value of α accelerates convergence and a smaller value of γ improves its robustness to measured noise. An investigation of the direct relation between the parameter variation in the EPDM family and the quality of images reconstructed by the proposed algorithm based on minimization of the EPDM is a future work to be considered. Moreover, we will use techniques such as machine learning to determine the most appropriate parameter depending on the noise level of the projections, number of projections, number of pixels, etc.

## Figures and Tables

**Table 1:** Values of the evaluation function for MLEM and PDEM with (γ,α) equal to (0.8,1.2) at 50th iteration, (0.5,1.2) at 100th iteration, and (0.3,1.2) at 200th iteration in the experiment using numerical phantom with noisy projection.

*N*	E(z(N))		
	**MLEM**	**PDEM with** (γ,α)	
50	6.44	6.29	(0.8, 1.2)
100	6.65	5.85	0.5, 1.2)
200	7.86	5.70	(0.3, 1.2)

**Table 2:** SSIM for MLEM and PDEM with the same parameters, as shown in Table 1 at *N*th iteration in the experiment using numerical phantom with noisy projection.

*N*	SSIM		
	MLEM	PDEM with (γ,α)	
50	0.651	0.689	(0.8, 1.2)
100	0.581	0.726	(0.5, 1.2)
200	0.531	0.772	(0.3, 1.2)

## Data Availability

All data used to support the findings of this study are included within the article.
